# Previous experience with delays affects delay discounting in animal model of ADHD

**DOI:** 10.1186/s12993-022-00199-z

**Published:** 2023-02-13

**Authors:** Espen Sjoberg, H. M. Ottåsen, R. G. Wilner, E. B. Johansen

**Affiliations:** 1 Kristiania University College, Prinsens gate 7-9, 0152 Oslo, Norway; 2 Oslo Metropolitan University, P.O. Box 4, St. Olavs Plass, 0130 Oslo, Norway; 3 University of Bergen, Sydnesplassen 7, 5007 Bergen, Norway

**Keywords:** ADHD, SHR, Animal model, Impulsivity, Delay discounting, Learning history

## Abstract

**Background:**

ADHD is a disorder where a common symptom is impulsive behaviour, a broad term associated with making sub-optimal choices. One frequently used method to investigate impulsive behaviour is delay discounting, which involves choosing between a small, immediate reinforcer and a delayed, larger one. Choosing the small immediate reinforcer is by itself, however, not sufficient for terming the choice impulsive, as all organisms eventually switch to choosing the small, immediate reinforcer when the delay to the larger reinforcer becomes long. This switch can be termed impulsive only when it occurs more frequently, or at shorter LL delays, than typically observed in normal controls. A poorly understood aspect is how choice is influenced by previous experience with delays. Using an animal model of Attention-Deficit/Hyperactivity Disorder, the Spontaneously Hypertensive Rat, we manipulated the order of exposure to delays in a delay discounting task. Following a preference test, the Ascending group experienced gradually increasing delays between choice and reinforcer while the Descending group were exposed to these delays in reverse order.

**Results:**

The results showed that the Descending group chose the small, immediate reinforcer over the larger delayed to a much larger extent than the Ascending group, and continued to do so even when the delay component was ultimately removed. Strain effects were found in the Ascending group, with SHRs switching to the small, immediate reinforcer earlier than controls as the delay to the larger reinforcer increased.

**Conclusion:**

The data suggests that delay discounting is affected by history of exposure to delayed consequences. When reinforcement contingencies are incrementally changed from having no response-reinforcer delay to a long delay, discounting of delayed consequences is gradual. However, a sudden change from no delay to a long delay, without intermediate training, results in a rapid switch to the small, immediate reinforcer option, and this behaviour is somewhat resilient to the shortening and eventual removal of the large reinforcer delay. The implication is that attempting to reduce already existing impulsive behaviour in children with ADHD will require gradual habituation and not sudden changes in reinforcement contingencies.

## Introduction

Attention-Deficit/Hyperactivity Disorder (ADHD) affects ~ 5% of the child population, and 2.5% of the adult [1–6], and exists in three subtypes: inattentive, hyperactive-impulsive, and combined [7]. A defining aspect of the combined and hyperactive-impulsive subtypes is impulsive behaviour, characterized as a tendency to act without foresight or making choices based on poor reasoning of future consequences [8]. However, the term “impulsive” is a broad label that classifies a range of traits, such as impatience, restlessness, risk or sensation-seeking behaviour, spontaneous or quick decisions, and lack of foresight [9, 10]. The terms impulsivity or impulsiveness hold a central place in psychological theories and psychiatric symptom’s lists, and their various operationalizations and neurobiological bases have been extensively studied. Due to the term’s heterogeneity and multifaceted nature, some have even argued that these concepts should be rejected and replaced with betted defined terms as they fail to meet the requirements of a psychological construct [11].

The exact cause of impulsive behaviour in ADHD is debated and depends on both the operationalization of the term and what theory one adopts [12, 13]. One theory of ADHD is the delay aversion theory, which proposes that impulsive behaviour is the result of an unwillingness to endure the temporal aspect of a choice, including the length between repeated choices [14]. An expansion of this theory is the dual component model of ADHD, which in addition to delay aversion incorporates a concept called impulsive drive for immediate reward (IDIR) [15]. This impulsive drive is the tendency for impulsive behaviour to be affected by the time between response and reinforcer for any given choice, specifically that longer response-reinforcer delays reduce the likelihood of it being chosen [15, 16]. Thus, the dual component model of ADHD suggests that both delay aversion and impulsive drive contribute together towards impulsive behaviour [15]. The dual component model does not explain the mechanism or processes behind IDIR but refers to other theories and explanations like executive dysfunction and deficits in inhibitory control, or that people with ADHD have a steeper delay-of-reinforcement gradient as suggested in the Dynamic Developmental Theory of ADHD (DDT, see [17]). The DDT offers detailed hypotheses regarding the behavioural and neurological mechanisms behind impulsive behaviour. It proposes that the effectiveness of a reinforcer is a decreasing function of the time between response and consequence, termed the delay-of-reinforcement gradient, and that this gradient is steeper in people with ADHD, meaning a steeper discounting of future reinforcers leading to the preference for small immediate reinforcers over large delayed [12, 17].

### Delay discounting

Delay discounting is a commonly used method for studying and measuring impulsive behaviour [18]. It usually involves choosing between a small reinforcer delivered immediately and a larger reinforcer delivered after a delay. All organisms eventually switch to choosing the immediate, small reinforcer as the delay to the larger reinforcer increases, despite the larger, delayed reinforcer being the theoretical option maximizing the amount of reinforcers in real time. Choosing the small, immediate reinforcer is adaptive in times of uncertainty (a bird in the hand is worth two in the bush), or during e.g., severe deprivation when immediate replenishment is needed for survival. Thus, as choosing the small, immediate reinforcer is sometimes normal or typical, impulsivity needs be defined relative to the choices of neurotypical controls [19]. Therefore, in this paper, we operationalize “impulsive” behaviour as when an organism significantly more often than neurotypical controls performs a choice leading to small reinforcers when large reinforcers are available at the cost of waiting and is the option maximizing amount of rewards in real time.

ADHD children, compared to controls, appear to display a reduced tendency to wait for a larger reinforcer and will typically choose the smaller option more often than neurotypical controls, i.e., they are impulsive [14, 20–23]. A meta-analysis suggests that people with ADHD are particularly sensitive to long delays, and are twice as likely as controls to make an impulsive choice in the presence of hypothetical reinforcers (e.g., points) compared to real reinforcers (e.g., money or food) in the task [24].

### The SHR animal model of ADHD

The Spontaneously Hypertensive Rat (SHR) is the most commonly used animal model of ADHD [25, 26], and largely considered the most validated model [27–30]. The rats were initially bred for high blood pressure research [31], but when compared to controls they exhibit similar characteristics to people with ADHD: they express impulsivity [32–35], inattention [28], hyperactivity [36], and increased behavioural variability [37, 38]. The SHR model is well researched, but has only been used a little more than a dozen times in delay discounting research [32, 35, 38–52]. Most studies on delay discounting using SHRs find that the rats act more impulsively on the task compared to controls [32, 35, 40, 42, 43, 45–47, 49, 50], indicated by a higher tendency to choose the small reinforcer when long delays are present for the large reinforcer, although other studies have failed to find any such strain difference [38, 41, 44, 48].

### The discrimination test and learning history in delay discounting

A discrimination test is a pre-experimental procedure where the animals are exposed to small and large reinforcers without delays, which purpose is to establish that the animal prefers the large over the small reinforcer option prior to any experimental manipulations. In other words, it is a test to verify that reinforcer size, and not operandum position or other variables, controls choice during no delay. This is a fundamental study requirement, as it is pointless to study choice as a function of delay to the larger reinforcer if reinforcer size does not control choice. Sjoberg and Johansen [19] emphasized the importance of including a discrimination test in order to avoid assumptions about the animals’ baseline preferences, but found that only three out of fourteen surveyed SHR studies clearly outlined the details of their discrimination test [39, 43, 51]. Three others specified the details but included a delay component during this phase [40, 41, 44], while the remainder either did not include such a test or did not specify the details involved. This reduces the possibility of direct comparison between studies.

In delay discounting, it can be argued that previous experience with the choice paradigm will influence the likelihood of a choice pattern occurring. An example of this is when animals are reused in a different experiment. For example, the rats used in Fox et al. [32] were later reused in a different delay discounting experiment by the same researchers [46], and the SHRs appeared to show a steeper discounting curve in the second experiment once a delay component was introduced. This observation alone, however, does not prove that previous experience was the cause, as a number of other factors may have influenced the results (e.g. the rats were also given saline or drug injections). In the SHR model of ADHD, only one previous study has examined the effects of learning history in delay discounting. Fox et al. [32] increased the delay between response and the large reinforcer in one condition, then subsequently reversed the order of the delays. The researchers found that SHRs relative to controls exhibited a greater preference for small, immediate reinforcers (small soon, SS) over larger, delayed reinforcers (larger later, LL) when delays were presented in descending order. The data showed that SS preference gradually increased along with increased delays for LL, but when this order was reversed the rats effectively maintained SS preference until the delay was almost absent. However, since this was a within-subject design, all the rats shared the same learning history, meaning that the results reflect a linear learning pattern where the rats adapt to increasing delays and then need time to readapt when these reinforcement contingencies are reversed. This suggests that once the rats are accustomed to delays, they require multiple repeated trials in order to readjust to short delays.

The current study aims to reproduce the experiment performed by Fox et al. [32], with certain adjustments. First, we will implement a lever preference test and assign the large later reinforcer to the lever least preferred. This will be followed by a discrimination test to ensure the rats discriminate between the small sooner (SS) and large later (LL) reinforcer. The rats must show a 66% or higher LL preference in two consecutive sessions before the experiment begins. This is similar to Fox et al. [32], who also used a discrimination test, but did not specify any criterion other than all rats preferring the large reinforcer “almost exclusively (p. 147)” by the end of the fourth session. Second, unlike Fox et al. [32], who used a within-subjects design, we will employ a between-subjects design. This means that, following the discrimination test, one group of rats will be exposed to gradually increasing delays (Ascending group) while another group will be exposed to these delays in reverse order (Descending group). Thus, the Descending group will be exposed to an abrupt and long delay to the large reinforcer and then decreasing delays as opposed to slow and gradually increasing delays in the Ascending group. Third, we will implement a procedure where the total trial length is constant and fixed at 24 s [19]. Therefore, as length of the delays change, inter-trial-intervals (ITIs) are adjusted to keep a constant trial duration. As a result, the two variables are always balanced and control for each other to the degree where one is absent, the other is at maximum (e.g., when delay is 0 s, ITI is 24 s). Fox et al. [32] also used a compensating design where the inter-trial interval would shrink in accordance with increased delays so as to assure that the trial lengths always remained constant [19, 53]. However, their inter-trial interval never disappeared completely. Finally, we will change the LL delay length between every daily session. This means that the animals will only be tested for 30 min at every delay condition, and no stable-state behaviour will be achieved. This will preclude the identification of pure reinforcer delay effects on LL choice, but has a larger ecological validity in terms of imitating naturally, rapidly changing contingencies and is also more like clinical testing in ADHD where one session of testing is the norm. Additionally, it has the advantage of showing the relative importance of learning history compared to reinforcer delay for the reinforcer sized used in the study.

Findings in previous studies of both animals and humans show that experience with increasing reinforcer delay can increase LL delay tolerance (e.g. [54–56]). In the Ascending condition in our experiment, LL delay is gradually increased, whereas in the Descending condition, LL delay is abruptly increased from 0 to 24 s. Therefore, without the gradual increase in LL reinforcer delay, we hypothesized that rats in the Descending condition will express steeper delay discounting and more SS choices compared to rats in the Ascending condition. Further, based on the results in Fox et al. (2008) and findings suggesting a steeper delay-of-reinforcement gradient in SHR/NCrl compared to WKY/NHsd [57–59], we expected to observe a higher percentage of SS choices and steeper delay discounting in SHR/NCrl relative to controls in both the Ascending and the Descending conditions.

## Methods

### Subjects

The study used 16 Spontaneously Hypertensive Rats from Charles River Laboratories, Germany (SHR/NCrl) and 16 Wistar Kyoto Rats from Envigo, United Kingdom (WKY/NHsd), all male and naïve at the start of the study. These specific strains were used because they have been argued to be the most appropriate model for ADHD [60]. The project was approved by the Norwegian Food Safety Authority, FOTS-ID 7994. The experiment was conducted at the Department of Biosciences, Blindern, University of Oslo.

The rats were five weeks old upon arrival (Day 1) and spent the next seven days habituating to their housing. This age was selected based on previous studies where the majority of experiments were conducted on rats between 5 and 12 weeks of age [38, 40, 41, 43, 44, 47, 49, 50]. Furthermore, an earlier pilot conducted by the same laboratory found that rats aged 3 weeks were often unable to exert enough force to close the micro-switch when pushing the levers in the chamber.

The rats were housed individually in 1290D Eurostandard Type III cages, 425 × 266 × 155 mm (820 cm^3^) raised wirelid series 123. Each cage contained a plastic tunnel, paper, and chew sticks (the latter two renewed weekly). The temperature was held stable between 18 and 22 degrees and measured daily along with humidity. The humidity was between 22% and 47% (except for one day when it was 63%), with an average of 32% throughout the experiment. The rats had a standard 12:12 day/night cycle, with lights on at 7 am and lights off at 7 pm. Experiments were conducted during the day cycle, Monday-Sunday. The rats had free access to food while in their cage, type 801,066 RM3(E) from Special Diet Service, England. The rats were weighted and handled weekly.

After the first day of habituation in the experimental chamber (Day 10), the rats were water deprived. From this point onwards they received water during the experiment and had one hour of free access to water immediately afterwards. Once the hour was up, water was taken away, and the rats were deprived for 22 ½ hours. The use of the 22 ½ -h water deprivation was justified by studies showing reduced learning effects for deprivation levels below 21 h [61], and that repeated daily 22-h water deprivation is minimally stressful and does not produce physiological changes [62]. During habituation, the average weights (in grams ± SEM) of the rats were 157 ± 2.8 and 104 ± 2.0, for SHR/NCrls and WKY/NHsds, respectively. During response shaping, the average weights were 182 ± 2.6 and 116 ± 2.9; at the start of the experimental phase they were 223 ± 2.7 and 167 ± 2.2; and at the end of the experiment weights were 234 ± 2.3 and 187 ± 1.8, respectively for SHR/NCrls and WKY/NHsds.

### Material

Experiments were conducted on four identical Campden 410-R boxes (25 × 21 × 20 cm), located at the Department of Biosciences, University of Oslo. The boxes had two retractable levers, a tray where food or water can be dispensed, along with three lights above the levers (not used) and a house light. The house light (20,7 lx) was on whenever the rat was in the chamber but was otherwise off. A small light inside the tray illuminated (21,2 lx) whenever a reinforcer was being delivered. The experimental program was made in Visual Basic 2010 Express. The data were saved both digitally as well as on a form filled out daily. Room-temperature water was used as the reinforcer.

### Design

The experiment was a 2 × 2 × 10 factorial design, with one within-subject variable (Delay condition, 10 days), and two between-subject variables (Strain and Order). The dependent variable was the percentage of responses producing the large reinforcer, while the independent variables were strain, delay condition, and order of delays. The data were analysed using ANOVA and *t*-tests, conducted in SPSS 24.

To avoid experimenter bias, the strain of the rats were blinded to the people conducting the study. A third party numbered all the rats prior to the start of the experiment and did not reveal the strain identity to the experimenters until data collection was complete.

## Procedure

### Habituation

On the 10th day after arrival, the rats were placed in the operant chamber for 30 min with the levers retracted and the house light on. Following this session, the rats were water deprived.

### Magazine training

On Days 11–13, the rats were subjected to magazine training. Here, a drop of water was delivered to the tray in the operant chamber according to a variable time (VT) reinforcement schedule, i.e. independently of the rat’s behaviour. These intervals were, in order, 20/20, 30/20 and 40/20. To clarify, an interval of 30/20 means that a reinforcer was delivered on average every 30 s +/- 20 s, i.e. the interval length varied between 10 and 50 s (range 40 s). During the first of these sessions, the lid shielding the water bowl where the reinforcers were delivered was taped open. For all subsequent sessions, the lid was closed, meaning the rats had to use their heads to open the lid in order to drink from the bowl.

### Shaping

Starting on Day 13, manual shaping of lever pressing began with the left lever. During the first day, each rat spent up to 60 min in the chamber, but this was reduced to 30 min on all subsequent days. Lever pressing was shaped according to the method of successive approximations; first, proximity to the lever was reinforced, then touching the lever, and finally pushing the lever. By the third day, stable lever pressing was established with all rats, and the rats produced 99.2% of all reinforcers delivered (the experimenters produced the remaining 0.8% as part of the training procedure). When shaping was switched to the right lever, all rats expressed stable lever pressing within two days.

### Preference – and discrimination test

Prior to conducting the discrimination test for the large reinforcer, we ran one lever preference test session (Day 19) where both levers produced one water drop. The purpose of this session was to determine if the rats held a response bias towards one lever over the other. For example, a rat may prefer the right lever, perhaps because it was further away from the chamber door or it was the last lever in the shaping procedure. If we then subsequently delegate the large reinforcer option to the right lever, this would be a confounding variable for, or bias toward, LL choices. In case of a 55% preference or higher for one lever over another, the rat was permanently assigned the opposite lever as producing the large reinforcer for the rest of the experiment. The lever preference test showed that 15 rats had a preference for the right lever (and were thus assigned the left lever for LL), 11 preferred the left lever, while the remaining six showed no preference and were randomly assigned a permanent LL lever.

During the discrimination test (Day 20–28), one lever produced five drops of water (LL) while the other produced one drop (SS – Small Sooner). The reinforcer size was determined by pumping time, where the mechanism pumping water into the tray ran five times longer for LL compared to SS. This meant that minor variations in reinforcer size occurred, but on average LL produced 0.35 ml of water, while SS produced 0.07 ml.

For each daily session, the rats were subjected to ten blocks of six trials. The first two of the six trials in a block were a forced choice trail: In these trials, each lever was presented alone (the program randomly determined which lever was presented first), giving the rat only one response option. The forced choice trials were included to ensure that behaviour would come into contact with the reinforcement contingencies (i.e. that the rats experienced the consequences of pressing both levers). After a response was made, an inter-trial interval (ITI) of 15 s occurred, during which time the levers were retracted into the wall, extending into the chamber again once the next trial began. The session ended when 60 trials had been completed, or when 30 min had passed, whichever came first. In order to pass the discrimination test, the rats needed to show a 66% preference for the LL option (or higher) two days in a row.

The discrimination test lasted nine days. Fifteen rats passed the test on their first attempt. By the fifth day, all but one rat had passed the 66% mark at least once, but there were signs of variation in many of the rats. By the ninth day, all the rats had passed the criterion except one that was marginally behind. However, it was decided to include the last rat because it had showed steady (albeit slow) progression and showed a 79% LL preference on the last day. During the experiment, this rat was monitored to see if its response pattern deviated from other rats in its group (it did not). There were no significant differences between strains in passing the discrimination test at any stage (all *p* > 0.05).

### Experimental phase

During the experimental phase (Day 29–38), the rats were split into two groups (Order variable): Ascending and Descending. The Ascending group was exposed to a delay between response and the LL reinforcer which increased systematically for each daily session, i.e. delay was increased from one day to the next. The delay was zero on the first day of the experimental phase, and this then increased in intervals of three seconds every session until a maximum of 24 s. We also added a one-second delay between the zero and three-second conditions, thus the sequence of delay intervals were 0, 1, 3, 6, 9, 12, 15, 18, 21, and 24 s. The LL was five times larger than the SS, and the SS option never had a delay. The trial length was fixed to 24 s, and the ITI for LL was adjusted in accordance with the delay in order to keep this constant. For instance, if the delay was 9 s, then the ITI was 15 s; when delay was 0 s, ITI was 24 s. The Descending group experienced the same setup as the Ascending group except that the order of delays was reversed. On the first day, they started with a delay of 24 s, which then gradually decreased across sessions.

Like during the preference and discrimination tests, each daily session consisted of ten blocks of six trials including two forced choice trails that ended when 60 trials had been completed or when 30 min had passed.

We set up *a priori* exclusion criterion: Any observation more than three standard deviations from the strain mean in the Ascending or the Descending groups would be excluded from that condition.

## Results

**Fig. 1:**
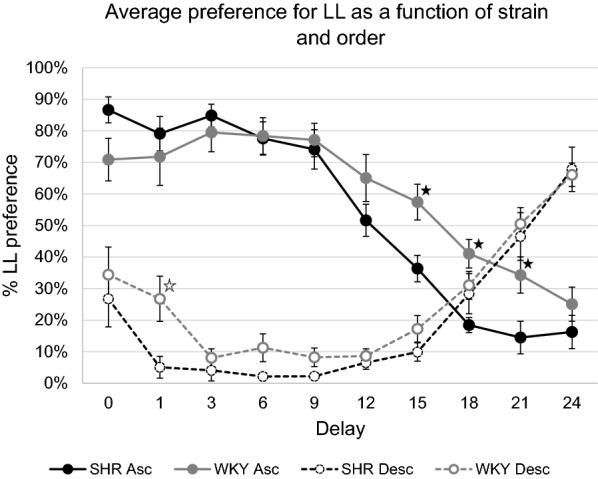
The average percentage of LL choices (Y axis) as a function of delay (X axis), strain (black = SHR, grey = WKY) and order (solid = Ascending, dotted = Descending). The stars indicate significant strain differences (solid star = Ascending, dotted = Descending). Error bars represent one standard deviation. There were 8 rats in each condition

Based on our *a priori* exclusion criterion, no data were excluded from the main analysis (only eight of 320 datapoints were two standard deviations away from their respective mean). The results are summarized in Fig. 1.

The 2 × 2 × 10 mixed ANOVA (with Bonferroni correction) found a main effect of Order, *F* (1,28) = 97.909, *p* < 0.0001, *η*_*p*_^*2*^ = 0.778, and of Delay, *F* (9, 252) = 13.103, *p* < 0.0001, *η*_*p*_^*2*^ = 0.319. There was no main effect of Strain, *F* (1,28) = 3.26, *p* = 0.082, *η*_*p*_^*2*^ = 0.104. In terms of interactions, there was a significant Delay x Order interaction, *F* (9, 252) = 99.237, *p* < 0.0001, *η*_*p*_^*2*^ = 0.78, suggesting that the Delay impacted the degree of LL preference for the rats differently for the two sequences. No statistically significantly Delay x Strain, *F* (9, 252) = 1.833, *p* = 0.063, *η*_*p*_^*2*^ = 0.061, nor Order x Strain, *F* (1, 28) = 0.001, *p* = 0.982, *η*_*p*_^*2*^ = 0.0001, interaction effects were found. However, there was a significant Delay x Order x Strain interaction, *F* (9, 252) = 3.926, *p* < 0.0001, *η*_*p*_^*2*^ = 0.123. This shows that Delay impacted the degree of LL preference differently across the two sequences, and that this pattern was different for the two strains. Follow-up *t*-tests for the statistically significant Delay x Order x Strain interaction effect, comparing LL choice for SHR/NCrls with WKY/NHsd in the Ascending or the Descending groups across the various delays, showed that only four out of 20 SHR/NCrl and WKY/NHsd comparisons were statistically significantly different. In the Ascending condition, SHR/NCrls had a higher proportion of SS choices at delay 15, *t* (14) = 2.984, *p* < 0.03, *d* = 1.492, delay 18, *t* (14) = 4.428, *p* < 0.001, *d* = 2.214, and at delay 21, *t* (14) = 2.561, *p* < 0.05, *d* = 1.28. There was only a significant strain difference at the 1 s delay point for the Descending group, *t* (14) = 2.721, *p* < 0.04, *d* = 1.364, where SHR/NCrls had a higher percentage of SS choices than controls. For all other comparisons, *p*s > 0.05.

## Discussion

Using a small-sooner over large-later delay discounting procedure, the current study tested the effect of delay exposure order in SHR/NCrl and WKY/NHsd controls. Two main research questions were studied. First, we tested the hypothesis that rats in the Descending delay condition exposed to an abrupt change from zero to long LL delays would express steeper delay discounting and more SS choices compared to rats in the Ascending delay condition where LL delays were gradually increased. These results should mirror the findings of Fox et al. [32], except that in the current study the preference switch would be a result of sudden rather than gradual changes in the response-reinforcer delays. Second, we expected to replicate the steepened delay discounting in SHR/NCrl relative to controls found in Fox et al. [32] and suggested in other studies [57–59], and tested whether the rapid change in LL delay in the Descending condition would increase strain differences.

While the results from the current study are not identical to those in Fox et al. [32], the studies complement each other and together paint a picture of learning curves and the influence and importance of previous experience in SHR/NCrl and WKY/NHsd controls. The curves obtained during the current Ascending condition replicated the curves observed in Fox et al. [32], and show a tendency for steeper delay discounting in SHR/NCrl relative to WKY/NHsd controls. The learning curves obtained from the Descending condition, however, were fundamentally different from the curves observed in the Ascending condition and those found in Fox et al. [32]. These Descending curves suggest that behaviour was heavily influenced by previous reinforcement conditions, and showed minimal strain differences in LL/SS choice.

### Differences between the Ascending and Descending delay conditions

The order of delay exposure profoundly affected percent choice of the large reinforcer in the current study. The Ascending group showed the expected pattern of a gradual decline in preference for the large, delayed reinforcer until a preference for the smaller reinforcer was established. By contrast, the rats in the Descending condition continued to choose the larger, delayed reinforcer, although to a smaller degree than during the discrimination test (average of 67% preference for the large reinforcer at the 24-second delay mark, compared to 89% in the final stage of the discrimination test). This is likely a hysteresis (carry-over) effect; the effects of the zero large reinforcer delay during the discrimination test continuing into the following 24-s delay condition. This is in line with what Sjoberg and Johansen [19] suggested, namely that many trials are required to establish the precise nature of a choice parameter. Once the Descending group in the current study switched to choosing the small reinforcers, LL preference never resurfaced, even when the delay was completely absent (only four of the 16 rats achieved an LL preference of 51% or higher when the delay was one or zero seconds), and in spite of the forced trials included at the start of each experimental sessions. The forced trials ensured that the rats gained experience with the consequences of choosing LL prior to the free trials, and constituted 1/3 of all trials each session. Still, the rats chose the LL most of trials in the 24-second delay condition and, conversely, chose the SS most of the trials when LL delays were absent or short. While the Descending group never reverted to choosing the large reinforcer, visual analysis of the data suggests a trend at the end of the experiment where the rats likely would have reverted to preferring LL with repeated exposure (Fig. 1). This again suggests that once the rats established a preference for SS, they required many trials with short or no delays before switching back to choosing LL.

In the second experiment in Fox et al.’s [32], rats were exposed to delay intervals in random order and for several sessions each delay condition. They found that the curves, likely resembling stable-state behaviour due to the many sessions used each condition, had the same general shape and was an intermediate between the curves found during the Ascending and the Descending conditions in their first experiment. A likely explanation for the different descending delay curves found in Fox et al. [32] and the current study is the study design. The current study used a between-group design where rats in the Descending condition had no experience with LL delay, whereas a within-group design was used in Fox et al. [32]. Here, the rats were first were exposed to LL delay in an ascending order before subjected to the descending order. Thus, the combined findings suggest that previous experience with reinforcer delay versus an abrupt change to long reinforcer delays can have a remarkable influence on behaviour and SS/LL preference.

### SHR/NCrl and WKY/NHsd comparisons

Similar to the findings in Fox et al. [32], comparisons of SHR/NCrl and WKY/NHsd in the Ascending condition showed a tendency for steeper delay discounting in SHR/NCrl, with the SHR/NCrl having a higher proportion of SS choices during delays 15–21 s relative to controls. In the Descending condition, however, no strain differences were found except during for delay 1 s where SHR/NCrl had a higher proportion of LL choices than WKY/NHsd. Findings in several studies indicate steeper delay discounting in SHR/NCrl relative to WKY/NHsd controls. Assuming these finding to be valid, the absence of strain differences in the Descending condition suggests that strain differences in delay discounting are overridden by the hysteresis effect. In the Descending 24 s and 21 s delay conditions, and for 120 trials including 1/3 forced trials, the rats chose the LL option 50% or more, suggesting that the continuing effects of previous reinforcement conditions has a larger influence on behaviour than reinforcer delay.

The SHR/NCrls in the current study had significantly more SS choices than WKY/NHsds. However, it should be noted that the effect of strain was only significant in the omnibus interaction analysis. The main effect of strain was not significant, although its effect size was moderate, *η*_*p*_^*2*^ = 0.104. Only four out of 20 strains comparisons were significant in the current study, and three of these occurred when the delay was above 15 s but became non-significant again at 24 s, possibly due to a floor effect. This may suggest that the SHR/NCrls develop SS preference at earlier than controls with increasing delays, but this pattern requires multiple trials before becoming evident, and it eventually plateaus to a floor effect and at this point strain differences can no longer be observed.

Compared to the literature, the strain differences observed represent mixed results. First, the SHR/NCrls in the current study had more SS choices than controls in the Ascending condition, similar to Fox et al. [32]. However, with increasing delays, the WKY/NHsd controls in the current experiment also switched preference from the large to the small reinforcer, while this was not the case in Fox et al. [32]. This may indicate a problem with the control group and not the SHR model itself, as previous studies have indicated that different vendor strains of WKY show genetic and behavioural differences, which is not the case with SHR [29, 54, 63], although it could also be due to methodological differences between studies. One such difference may be the type of reinforcer used. The current study used water reinforcers whereas e.g., Fox et al. [32] used food pellets. Whether results can be generalized across reinforcer types requires further investigation.

### Limitations

The current results support the findings of Fox et al. [32] and suggests that previous experience plays an important role in delay discounting. However, certain limitations should be addressed, particularly when comparing the study to that of Fox et al. [32]. First, other than differences in the experimental manipulations already outlined, the current study used naïve rats while those in Fox et al. [32] had previous experience, although not in delay discounting. This also means that their rats were older: approx. eight months old at the start of the experiment compared to just over a month old in the current study. Second, our strains were from European vendors while Fox et al.’s [32] were American. Third, in the current study the delay component increased or decreased by three seconds for every session (except for when the delay was one second). By contrast, Fox et al. [32] used a doubling-procedure where the delay was first three seconds, then six, 12 and finally 24. This means that the gaps between the delays were larger, and arguably the rats therefore had less time to adapt to the changes in delay compared to the current study. This could account for why the WKYs expressed a preference switch in the current study but not in Fox et al. [32], considering that we effectively doubled the number of sessions. Nevertheless, this could also be due to vendor strain differences, as the SHR results are otherwise similar, suggesting that while the interval method in the current study paints a more linear picture, it most likely did not significantly affect the result (at least not for SHRs). There were also other minor differences of note: Fox et al. [32] used pellets, with LL being five times larger than SS, and did not use retractable levers. The current experiment used water, with LL being five times larger than SS, and levers retracted following a response.

## Conclusion

The current study aimed to investigate the effect of previous experience on delay discounting in an animal model of ADHD, more specifically how the order of delays affects SS over LL preference. It was found that a sudden and drastic change in delay parameters, going from no delay to a long delay, produced SS preference in both SHR/NCrls and controls, a behaviour that never reverted even when the LL delay was short or absent. By contrast, the more common ascending procedure, where delays gradually increase of the course of the experiment, showed that both SHR/NCrls and control switch preference from LL to SS once the delay passes a certain threshold, roughly 12–15 s in the current experiment. These findings suggest that previous exposure to delays will affect performance on delay discounting tasks, and that both SHR/NCrls and controls require a substantial number of trials in order to re-establish LL preference once the delay components have been reduced or removed. The implication for people with ADHD is that sudden changes in reinforcement schedules may not show immediate effects but require either a gradual adaptation or exposure to multiple trials.

## Data Availability

Raw data file available upon request.
